# Damage and Recovery Behavior of Low-Replacement-Rate Fly Ash Concrete after Different High-Temperature Exposures

**DOI:** 10.3390/ma17174330

**Published:** 2024-08-31

**Authors:** Lin Mi, Bowen Kuang, Daixin Fu, Lang Li, Yongjie Liu, Chong Wang, Chao He, Yao Chen, Hong Zhang, Fulin Liu, Qingyuan Wang

**Affiliations:** 1 Failure Mechanics and Engineering Disaster Prevention Key Laboratory of Sichuan Province, College of Architecture and Environment, Sichuan University, Chengdu 610207, China; scumilin@stu.scu.edu.cn (L.M.); kuangbw@email.cn (B.K.); fudaixinscu@outlook.com (D.F.); liuyongjie@scu.edu.cn (Y.L.); wangchongscu@163.com (C.W.); hechao@scu.edu.cn (C.H.); yaochen@scu.edu.cn (Y.C.); zzhanghong@scu.edu.cn (H.Z.); liufulin@scu.edu.cn (F.L.); wangqy@scu.edu.cn (Q.W.); 2 MOE Key Laboratory of Deep Earth Science and Engineering, College of Architecture and Environment, Sichuan University, Chengdu 610065, China

**Keywords:** high-temperature exposure, post-fire curing, fly ash, microstructure, compressive strength

## Abstract

This study focuses on investigating the strength recovery of fire-damaged fly ash concrete (FAC) with a low substitution rate of 10% through post-fire curing. The chemical and microstructural changes were analyzed using X-ray diffraction (XRD), derivative thermogravimetry (DTG), scanning electron microscopy (SEM), energy dispersive X-ray spectrometry (EDS), and nitrogen adsorption. The findings indicate that the incorporation of fly ash slightly enhanced the strength after exposure to 400 °C; this was attributed to improved pozzolanic reactions, which were not observed at higher temperatures of 600 °C and 800 °C. Moreover, a positive effect on the recovery of compressive strength was observed due to the pozzolanic reaction. However, due to the relatively low fly ash content, depletion occurred at a later age, resulting in the inability to inhibit microstructural damage caused by the production of portlandite, thereby weakening the compressive strength. Interestingly, fly ash influenced the morphology of calcium carbonate and calcium silicate hydrate crystals, which is potentially ascribed to the role of high aluminum content acting as a crystallization-guiding agent.

## 1. Introduction

Concrete, one of the most widely used building materials globally, is susceptible to fire damage. While concrete is generally considered a fire-resistant material compared with steel or wood, it experiences significant physical and chemical changes when exposed to high temperatures, leading to the degradation of its service properties [1,2,3,4,5,6]. Typically, the deterioration triggers arising from high-temperature exposure can be classified into three main categories: thermal mismatch between different phases in concrete, pore pressure development, and decomposition of hydrates [7]. When exposed to high temperatures, the differential deformation between the contracting hardened cement pastes and expanding aggregates, along with the non-uniform temperature distribution, induces thermal stress, resulting in subsequent microstructural deterioration [8]. The evaporation of pore water during high-temperature exposure leads to the coarsening of the pore structure due to the tensile strength induced by increasing steam pressure. However, certain literature studies have also reported strength gains in concrete exposed to temperatures below 300 °C. These gains are attributed to the accelerated hydration process, which correlates with the internal autoclaving condition induced by the evaporation of pore water [9,10,11]. The decomposition of various hydrates, including ettringite, monosulfate, calcium silicate hydrates (C-S-H), portlandite, and calcium carbonate, has been extensively investigated. Ettringite and monosulfate dehydrate within the temperature range of 60–150 °C, while unhydrated calcium silicate (C_n_S) transforms into C-S-H gel below 400 °C under autoclaving conditions [11,12,13]. The C-S-H gel undergoes dehydration, losing bond water at 100–300 °C, and decomposes into C_n_S at 450–800 °C [12,14,15]. The dehydration of portlandite initiates at 300 °C, accelerates, and concludes at 400–550 °C, while calcium carbonate decomposes within the temperature range of 650–1000 °C [9,14,16,17]. It is noteworthy that compared with ordinary Portland cement, the C_n_S in dehydrated Portland cement exists more as C_2_S than as C_3_S [18,19].

In the realm of concrete post-fire repair, the aftermath of high-temperature exposure often necessitates remedial interventions. An array of methods exists for restoring fire-damaged concrete structures. These include section enlargement strengthening [20], the application of post-compressed steel plates [21], or the removal of compromised concrete segments followed by replacement with fresh concrete [22]. However, these conventional repair techniques typically entail substantial quantities of extra materials, including high-performance concrete [23], resin, or steel, thereby incurring comparatively higher economic costs when compared with the simplicity of water-based recuring methods.

Interestingly, the existing literature has highlighted that restoration through water recuring stands on par with, or may even surpass, the efficacy of these resource-intensive alternatives [24]. Despite the property deterioration following high-temperature exposure, concrete can be repaired through the rehydration of dehydrated compounds with sufficient water [25,26,27,28]. C_n_S rehydrates to C-S-H gel, and CaO rehydrates to portlandite. The rehydration process helps restore concrete properties by filling the thermal-induced voids. However, it is important to note that the rehydration of CaO to portlandite results in a significant 97% volume expansion, leading to further microstructural damage [29,30]. Furthermore, the hydration process of C_2_S generates CaO, which subsequently reacts with water to form portlandite [31,32]. This additional formation of portlandite can contribute to non-negligible expansion [19].

Fly ash, a silica- and aluminum-rich supplementary cementitious material, is widely utilized in building construction due to its beneficial impact on concrete properties, economic advantages, and environmentally friendly nature [33]. Extensive research has been conducted to investigate the effect of fly ash on the damage to and rehydration process of high-temperature exposed concrete, with most studies reporting a positive influence on the recovery of post-fire cured concrete [26,34,35,36,37]. Poon et al. [26] compared concrete with a 30–40% fly ash replacement to conventional concrete after exposure to temperatures of 600 °C and 800 °C followed by rehydration. Their findings revealed that the unhydrated fly ash present in the concrete reacted with the portlandite formed during the rehydration process, resulting in the formation of C-S-H gel. This reaction, known as the pozzolanic reaction, is attributed to the high silica content of fly ash. The newly formed C-S-H gel filled the capillaries, enhancing the microstructure of the concrete and consuming portlandite to prevent the formation of expansion-induced cracks during the rehydration process. Shui et al. [37] subsequently confirmed these findings using various test techniques.

Previous studies have predominantly focused on high (≥30%) fly ash replacement concrete and reported a consistently favorable recovery of macroscopic properties. However, it is important to note that *Specification for mix proportion design of ordinary concrete JGJ 55-2011* restricts the replacement rate of fly ash to less than 35% and limits it even more, to less than 30%, in low-water–cement-ratio concrete [38]. Furthermore, the specification also mandates a soundness test for fly ash replacement above 30%. Consequently, lower fly ash replacement rates are preferred in most construction projects. In this study, we investigated the strength recovery during the recuring process of mortar with 10% fly ash replacement and compared it with that of plain mortar. Surprisingly, our findings did not show a consistently continuous strength recovery process, as previously reported. To elucidate the underlying mechanisms, a variety of characterization methods, including X-ray diffraction (XRD), derivative thermogravimetry (DTG), scanning electron microscopy (SEM), energy dispersive X-ray spectrometry (EDS), and nitrogen adsorption tests, were employed to analyze the microstructure and chemical component evolutions. The outcomes of this study can significantly contribute to the estimation of the post-fire curing properties of low-substitution-rate fly ash concrete (FAC).

## 2. Materials and Methods

### 2.1. Raw Materials and Mix Design

The binder materials used in this study consisted of P.C. 42.5R composite silicate cement that aligned with the specifications detailed in *GB 175-2023 Common Portland Cement* [39], produced by the Lafarge Group, and fly ash. The chemical compositions were determined by X-ray fluorescence (XRF) using the Rigaku ZSX Primus III+. Fine aggregate was obtained from ISO Standard Sand CO. Ltd., Xiamen, China, and conformed to ISO standards. The grading curve of the fine aggregate was tested according to *GB/T 14684-2011 Sand for Construction* standards [40]. Mortar specimens were prepared using the mix ratio presented in Table 1 based on the suggested mix ratio from *GB/T 17671-2021 Test method of cement mortar strength (ISO method)* and *ISO 679:2009—Cement—Test methods—Determination of strength* [41,42]. The mortar was blended following the procedure outlined in *GB/T 17671-2021*. Subsequently, the mixture was cast into steel molds, and the specimens were cured in the atmosphere for 24 h before demolding. After demolding, the specimens were appropriately labeled and transferred to a water tank for a curing period of 90 days before being subjected to testing.

### 2.2. High-Temperature Exposure and Recuring

**Figure 1:**
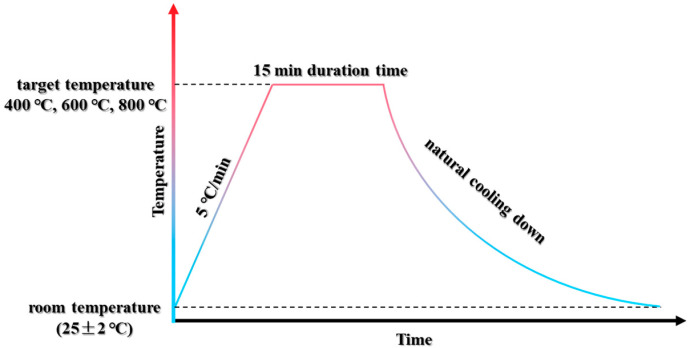
Schematic of the specimen high-temperature exposure procedure.

After a 90-day curing period, the specimens were placed in the laboratory environment for 7 days to allow for water drying; this was aimed at preventing spalling. Subsequently, an electrical high-temperature furnace was employed to subject the specimens to elevated temperatures, following the temperature–time procedure depicted in Figure 1. Initially, the specimens were heated at a consistent rate of 5 °C/min until the target temperatures of 400 °C, 600 °C, or 800 °C were reached. Once the target temperature was reached, it was maintained for 15 min. Following the heating and cooling process, three specimens from each group were selected for immediate testing, while the remaining specimens were subjected to post-fire curing in water for 28 and 60 days, respectively. Specimens exposed to different temperatures were immersed in separate tanks to ensure proper post-fire curing conditions.

### 2.3. Test and Sampling Methods

**Figure 2:**
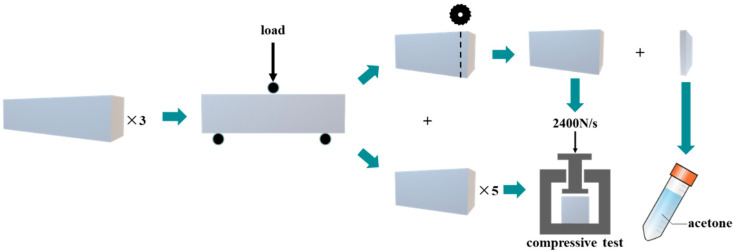
Schematic of macro-test regimes of the specimens.

The macroscopic tests were conducted following the procedure outlined in Figure 2. The specimens were flexed into two parts, which were subjected to different treatment conditions, including 90-day regular curing, recuring after the set age, or high-temperature damage. To ensure statistical reliability, a minimum of three specimens were tested for each mix and temperature condition at each stage. For the microstructural analysis, one section with a thickness of 8 mm was sliced off from one of the six flexed parts before conducting the subsequent compressive test. The remaining five flexed parts, along with the section preserved for microstructural analysis, were utilized for the subsequent compressive test.

The sliced piece was immersed in acetone to prevent further hydration until it was ready for microstructure testing. Prior to observation, the samples underwent a polishing process using a polishing machine set at a speed of 180 rpm. Sandpapers with mesh grades of 240, 600, 1000, 1500, and 2000 were used, followed by polishing with a 2.5 μm diamond abrasive. Subsequently, the samples were washed with clean water and dried using compressed air. However, some specimens exposed to relatively high temperatures suffered severe damage, rendering them unsuitable for conventional polishing. Therefore, a cold inlay technique using epoxy resin was employed to protect their microstructures. This process involved embedding the damaged specimens in epoxy resin to facilitate the subsequent microstructural analysis. A Hitachi TM3000 desktop SEM and Zeiss Sigma 300 field-emission SEM with a Bruker XFlash 6130 EDS were used to obtain backscattered electron (BSE) and secondary electron (SE) images, respectively, as well as for the elemental analysis.

To analyze the microstructural changes, XRD and thermogravimetric analysis–derivate thermogravimetric (TGA-DTG) techniques were employed to explore the chemical influences on the microstructure variations among the two groups before and after high-temperature exposure and recuring. Test samples were acquired by crushing the sliced samples and then grinding them into fine powders of less than 75 × 10^−3^ mm in size. XRD scans were conducted using a Rigaku Ultima IV instrument equipped with monochromatic Cu-Kα (λ = 1.54056 Å) radiation with a scan speed of 2°/min and a step size of 0.02046° in a 2θ range of 15–55°. The TGA curves were generated using a NETZSCH 449F3 instrument under a nitrogen atmosphere at a heating rate of 20 °C/min from 25 °C to 1000 °C.

Unlike AFt/AFm, the decomposition temperature interval of portlandite (370–500 °C) hardly overlaps with the weight-loss temperatures of other substances. As a consequence, the portlandite content can be quantitatively analyzed based on DTG data [43]. The content of portlandite (CH) can be calculated according to the equation below and expressed as a % of the dry sample weight [43,44,45]:

$$CH\%=\frac{w_{500}-w_{370}}{w_{370}}\times \frac{74}{18}$$

where $w_{i}$ refers to the sample weight at i °C.

In addition, nitrogen adsorption tests were performed using Micromeritics ASAP 2460 to obtain the specific surface area of the specimens during the recuring process. The sampling method and measurements were the same as those reported in the literature [46].

## 3. Results and Discussion

### 3.1. Raw Materials

**Figure 3:**
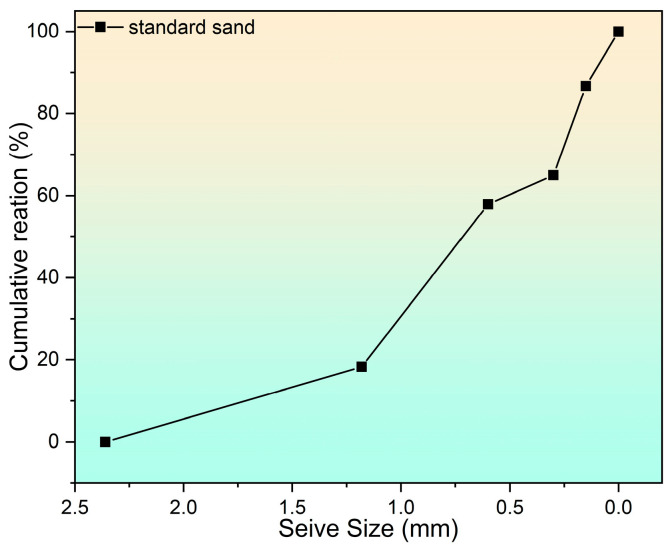
Grading curve of the fine aggregate.

The chemical compositions of cement and fly ash can be found in Table 2, which indicates the high calcium content in the cement as well as the silica- and aluminum-rich properties of the fly ash. The grading curve of the fine aggregate is shown in Figure 3.

### 3.2. Flexural and Compressive Tests

The data obtained from previous studies consistently showed a steady recovery of the concrete compressive strength during the recuring process. However, in this study, the concrete compressive strength exhibited varying degrees of fluctuation during recuring, particularly in the case of concrete exposed to temperatures of 400 °C and 600 °C. Considering the variations in initial strength among these groups, a comparative measure of recovery, termed the relative compressive strength (RCS), was proposed. The RCS represents the ratio of the strength at different temperatures and stages relative to the strength after 90 days of regular curing. The residual compressive strength of the specimens exposed to high temperatures and subjected to recuring, as well as their corresponding RCS values, are presented and discussed below.

**Figure 4:**
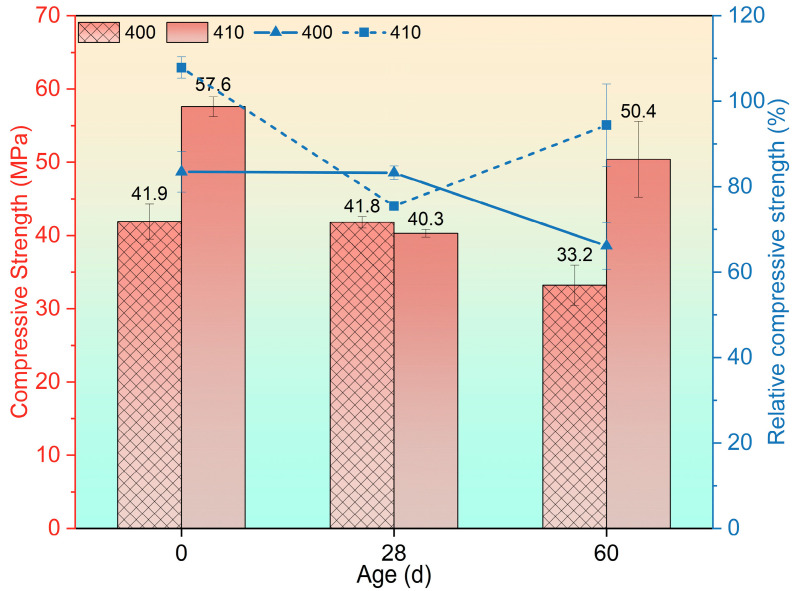
Residual compressive strength after being exposed to 400 °C and recuring.

The effect of fly ash incorporation on compressive strength after exposure to 400 °C at different recuring ages is illustrated in Figure 4. Upon exposure to 400 °C, specimens containing 10% fly ash replacement exhibited a higher relative compressive strength (RCS) of 107.86% compared with those without fly ash. This slight increase in post-high-temperature concrete strength has been reported in previous studies, which attributed the phenomenon to the autoclaving conditions induced by water evaporation [47,48,49]. However, in the case of the 410 group, the compressive strength decreased after a 28-day recuring but recovered to 50.4 MPa (RCS = 94.38%) by the 60-day recuring age. For the 400 group, the compressive strength reduced to 41.9 MPa (RCS = 83.47%), without significant changes observed after the 28-day recuring. However, a further strength reduction to 33.2 MPa (RCS = 66.14%) was observed after the 60-day recuring. Notably, the strength of the 410 group was 37.47% and 51.81% higher than that of the 400 group after exposure to 400 °C and following a 60-day recuring, respectively, while no significant difference was observed after the 28-day recuring.

**Figure 5:**
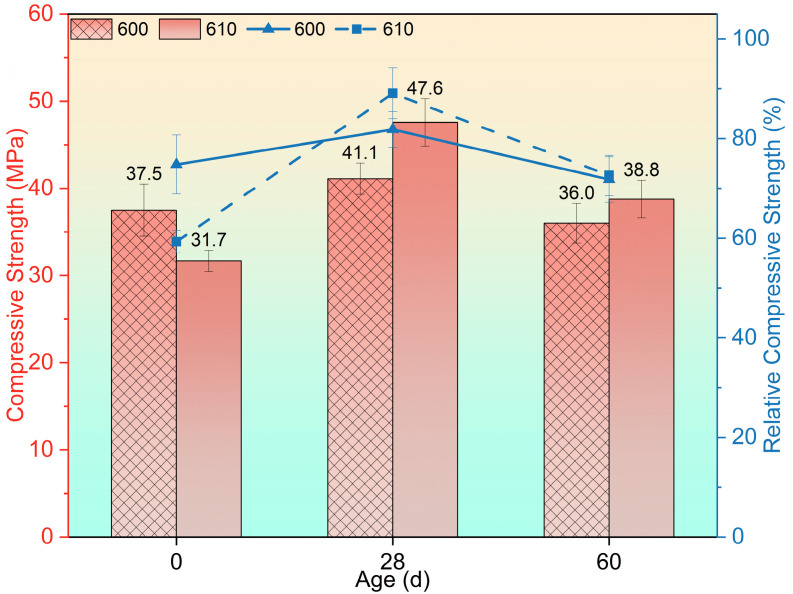
Residual compressive strength after being exposed to 600 °C and recuring.

The compressive strength of group 600 and group 610 following high-temperature exposure and recuring is presented in Figure 5. Despite exhibiting lower strength after exposure compared with group 600, group 610 demonstrated a faster strength recovery up to the 28-day recuring age. Notably, both group 600 and group 610 experienced strength reductions of 12.41% and 18.49%, respectively.

**Figure 6:**
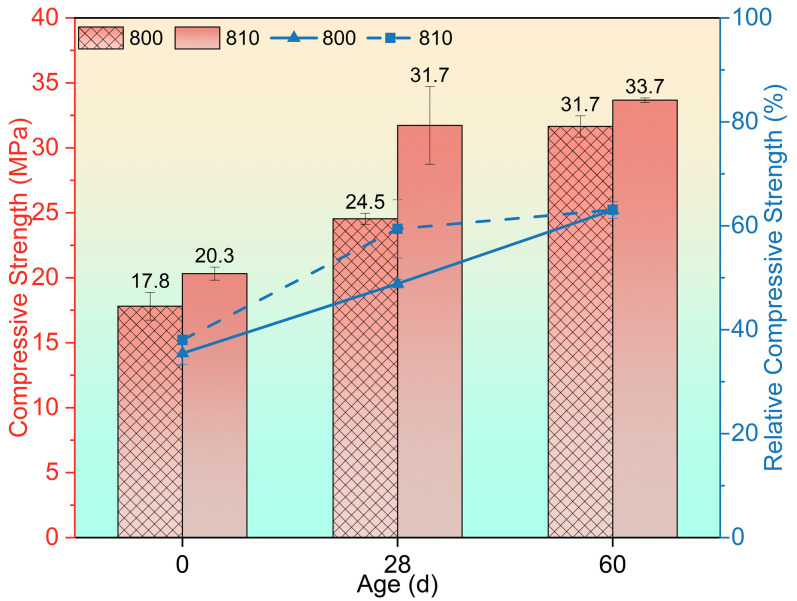
Residual compressive strength after being exposed to 800 °C and recuring.

The compressive strength after exposure to 800 °C and recuring is presented in Figure 6. Following the 800 °C exposure, the strength reduced to 17.8 MPa (RCS = 35.48%) and 20.3 MPa (RCS = 38.06%). Continuous strength recovery then occurred in both groups with the increase in recuring age. Notably, at the 28-day recuring age, group 810 demonstrated a faster strength recovery (31.7 MPa, RCS = 59.42%) compared with group 800 (24.5 MPa, RCS = 48.89%). However, the strength of the 810 group was not higher than that of the 800 group at the 60-day recuring age.

### 3.3. Microstructural and Chemical Composition Studies

#### 3.3.1. XRD Studies

**Figure 7:**
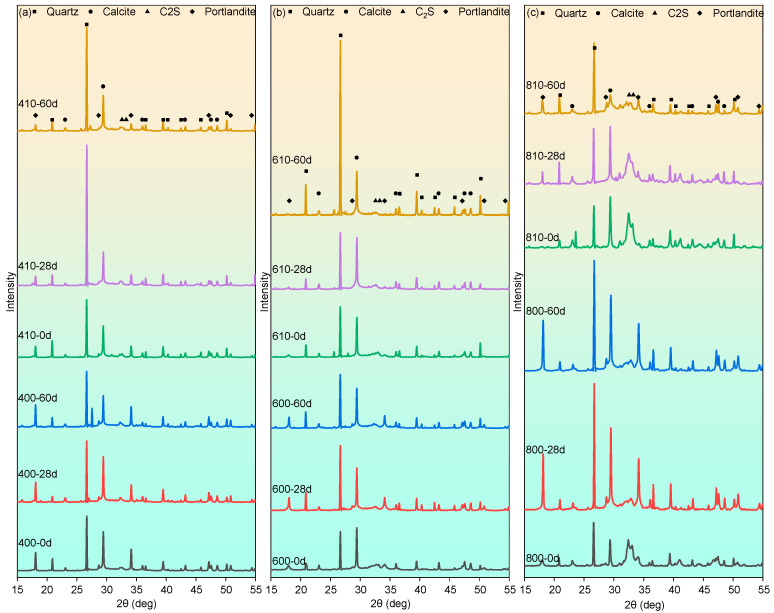
XRD patterns of mortar samples after varying high-temperature exposure and recuring: (**a**) 400 °C, (**b**) 600 °C and (**c**) 800 °C.

The phase analysis of mortar samples by X-ray diffraction (XRD) is presented in Figure 7. The XRD pattern reveals the presence of typical hydrated phases, such as portlandite and calcium carbonate (calcium carbonate), and dehydrated phases such as C_2_S. The presence of quartz is also observed due to the utilization of siliceous sand in the concrete mix. It can be observed that the C_2_S content increased with higher temperatures during high-temperature exposure and decreased during the recuring process, especially in samples after the 800 °C exposure. Additionally, a high content of calcium carbonate is evident from the peak at 2θ = 29.405° in all the groups, which can be attributed to the calcium-rich nature of the cement used. However, the intense spectral line corresponding to quartz (2θ = 26.639°) significantly interferes with the accurate assessment of the content of other substances. Moreover, it is important to note that XRD patterns are more suitable for qualitative or semi-quantitative analyses, and thus, the intensity of these spectral lines may not precisely represent the differences in the content of various substances between the groups. Hence, the specific substance content differences will be discussed based on the results of the thermogravimetric analyses presented below.

#### 3.3.2. Thermogravimetric Analysis

**Figure 8:**
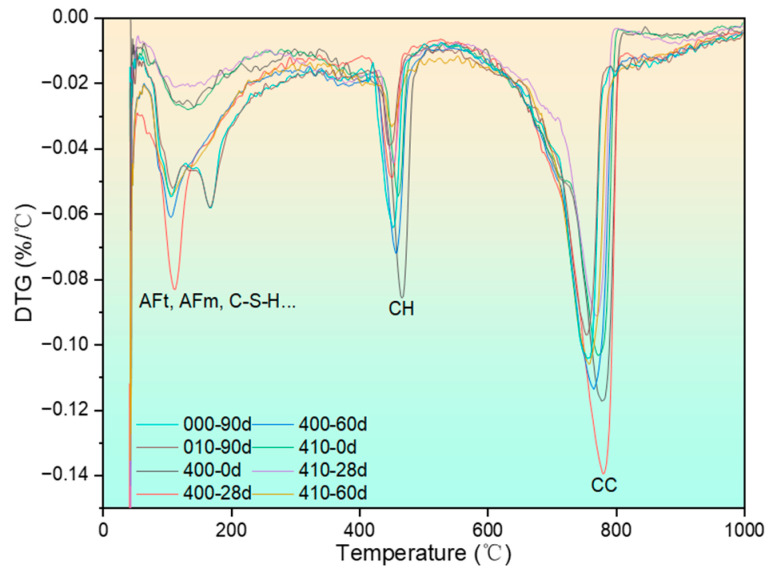
DTG curves of specimens after being exposed to 400 °C and recuring.

The differential thermogravimetry (DTG) curves of specimens after exposure to 400 °C and recuring are presented in Figure 8. Due to the large mass of the test samples, some temperature hysteresis effects are observed across the curves. However, these effects do not hinder the identification of peaks corresponding to different substances since the peaks are well separated. Previous studies have identified the substances represented by the individual peaks on the DTG curve, which have been appropriately labeled in the figure [43].

**Figure 9:**
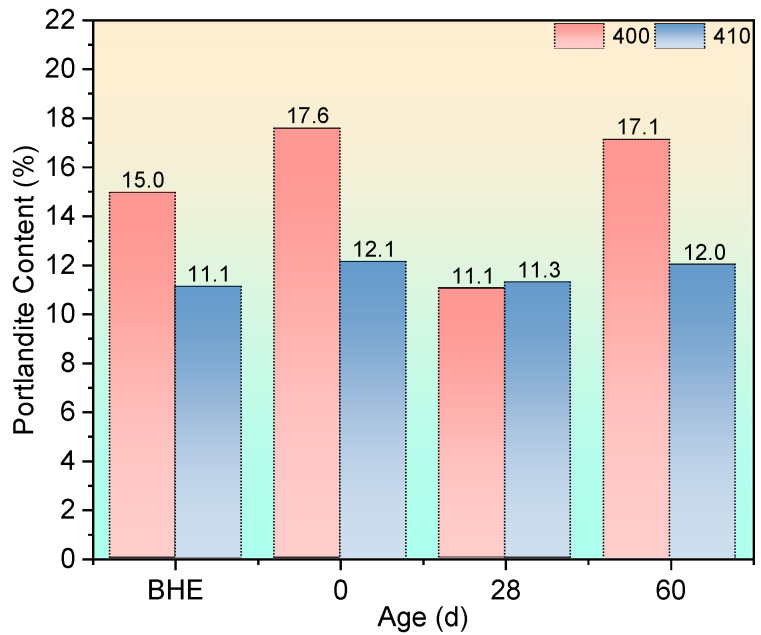
Portlandite content after being exposed to 400 °C and recuring (BHE: before high-temperature exposure).

The results of the quantitative analysis of portlandite are shown in Figure 9.

In Section 3.1, a comparison between group 400 and group 410 revealed contrasting effects of the autoclaving condition on strength changes after high-temperature exposure. As shown in Figure 9, both group 400-0d and group 410-0d exhibited an increase in portlandite content due to the accelerated hydration process facilitated by autoclaving. However, the outcomes differed between the two groups. Following high-temperature exposure, group 400 displayed a significantly higher increment in its portlandite content (17.3%) compared with group 410 (9.1%). This discrepancy can be attributed to the consumption of portlandite by fly ash in group 410, which possessed pozzolanic reactivity, thereby preventing cracking through expansive crystallization. In contrast, group 400 lacked an effective mechanism to mitigate this damage. Consequently, these micro-scale mechanisms influenced macro-scale impacts, resulting in divergent strength changes between the two groups.

As the recuring process progressed, a notable decline in portlandite content was observed in group 400 at the 28-day recuring age. This reduction was attributed to the dissolution of significant amounts of portlandite through the water-immersed recuring method. In contrast, the autoclaving condition led to a denser microstructure in group 410 compared with group 400, limiting water penetration through capillaries and thus inhibiting portlandite dissolution. At the 60-day recuring age, the portlandite content in group 400 mortar specimens experienced a sharp increase as the dissolution of portlandite in water reached saturation. Consequently, the strength of group 400 at the 60-day mark decreased. On the other hand, in group 410, the rising portlandite content in water facilitated further reactions between fly ash and portlandite in the surface layer, resulting in enhanced strength and a denser microstructure. Detailed microstructural evidence supporting these findings will be presented in Section 3.3.3. Additionally, the cause of strength decay in group 410-28d will be explained in Section 3.3.3 and supported by SEM evidence.

**Figure 10:**
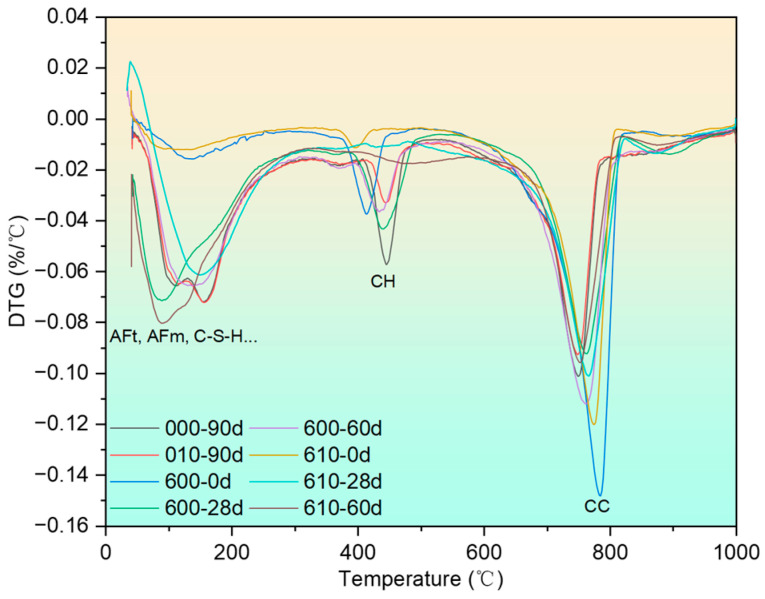
DTG curves of specimens after being exposed to 600 °C and recuring.

**Figure 11:**
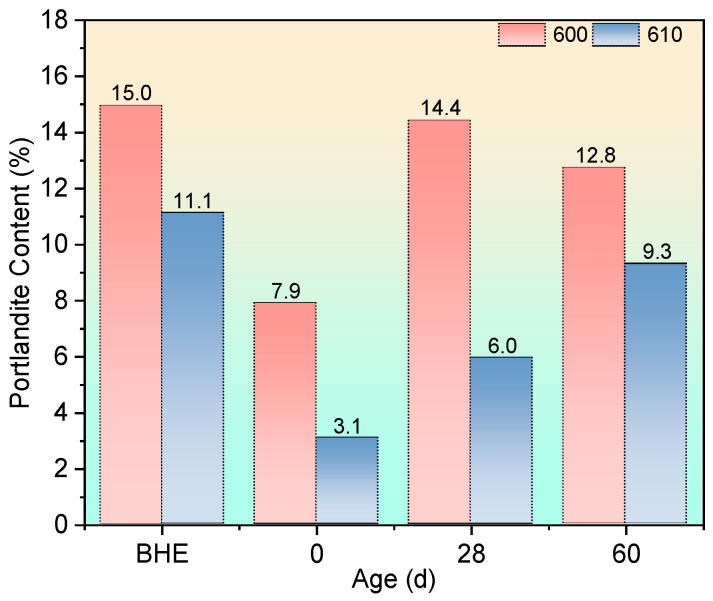
Portlandite content after being exposed to 600 °C and recuring.

Figure 10 shows the DTG curves of specimens after being exposed to 600 °C and recuring. Following calculations based on the abovementioned equations, the portlandite content is shown in Figure 11.

After a 600 °C exposure, it is commonly observed that the portlandite content decreases due to its dehydration temperature being around 450 °C. Additionally, high-temperature exposure has been reported to promote carbonation in concrete, which can contribute to the reduction in portlandite content and explain the increase in calcium carbonate content following a 600 °C exposure [50,51].

During the recuring process, the portlandite content of group 600 increased by 6.5 percentage points at 28 days compared with after high-temperature exposure, while group 610 showed a 2.9 percentage point increase at the same age. This disparity can be attributed to the pozzolanic reactivity of fly ash, which depletes the portlandite and protects the microstructure. Consequently, this explains the faster strength growth observed in group 610 compared with group 600 at 28 days.

Furthermore, despite a noticeable increase in portlandite content during the initial 28 days of recuring, the strength exhibited slow growth. This phenomenon can be attributed to two main factors. Firstly, the damage caused by high-temperature exposure creates ample space for crystal growth, thereby preventing cracking from expansive crystallization. Secondly, the rehydration of calcium silicate plays a more significant role in the recuring process following exposure to 600 °C compared with 400 °C.

However, it should be noted that more C_2_S formed after the 600 °C exposure compared with the 400 °C exposure due to more severe dehydration. The hydration of C_2_S leads to the formation of calcium oxide, which further undergoes hydration to form portlandite. Consequently, a larger amount of fly ash is consumed in this process. As a result, by the time the 60-day recuring age after 600 °C high-temperature exposure is reached, the fly ash tends to be fully reacted and is unable to further inhibit portlandite formation. In group 610, where the exposure temperature is 600 °C, the microstructure of the mortar becomes highly dense. This dense microstructure limits the available space for portlandite crystallization during the later stages of recuring. Consequently, a decrease in macroscopic strength, attributed to microstructural deterioration, is observed in the 60-day age samples.

**Figure 12:**
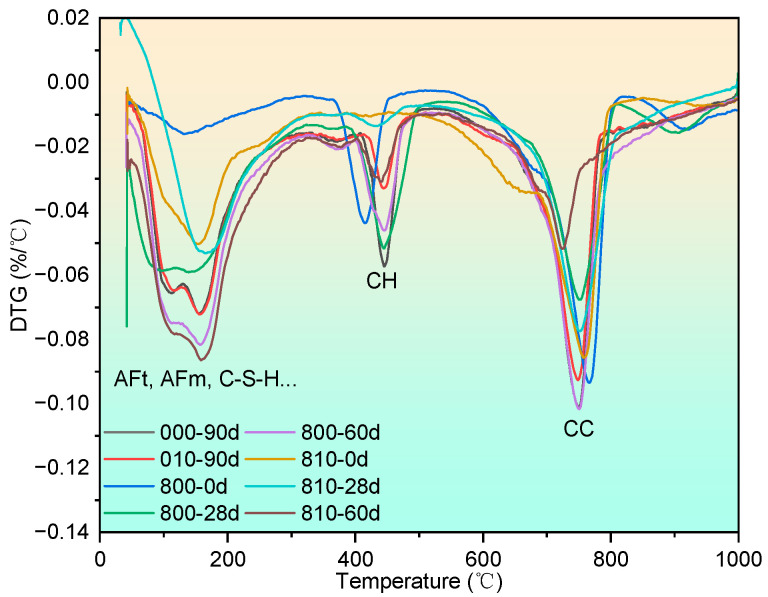
DTG curves of specimens after being exposed to 800 °C and recuring.

**Figure 13:**
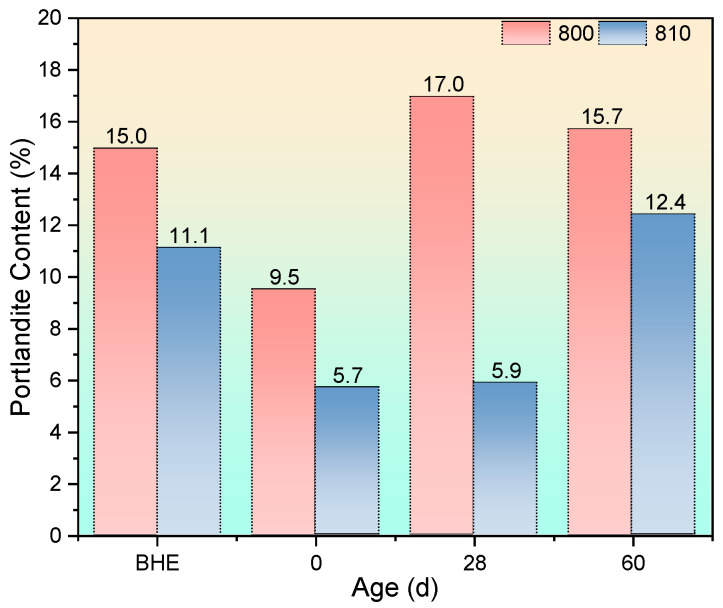
Portlandite content after being exposed to 800 °C and recuring.

The complete DTG curve can be seen in Figure 12, and the portlandite content after being exposed to 800 °C can be seen in Figure 13.

After exposure to 800 °C, the portlandite content in both groups of mortar samples decreased, similarly to the effects observed after 600 °C exposure, which can be attributed to high-temperature decomposition and carbonation. Subsequently, the portlandite content of the group 800 samples increased by 7.5 percentage points after 28 days of recuring, while the portlandite content of the 810 samples remained relatively unchanged. Although expansive crystallization of portlandite has been recognized as a primary factor negatively affecting the strength recovery of specimens exposed to 400 °C and 600 °C, there is insufficient microstructural evidence to support this claim. However, after an 800 °C exposure, significant voids emerged, providing ample space for portlandite formation, particularly during the early recuring stage (within 28 days). The lower strength recovery observed in the 800 group, compared with the 810 group, can be attributed to differences in microstructure density resulting from varying calcium carbonate morphologies. Furthermore, at the 60-day recuring age, a substantial increase in portlandite content was observed in the 810 group due to the enhanced rehydration of C_2_S compared with the 610 group, resulting in greater portlandite production. This, combined with the relatively dense microstructure, led to the increment of microcracks, influencing the strength recovery. Detailed microstructural evidence supporting these observations will be presented in Section 3.3.3.

#### 3.3.3. SEM Observation and the EDS Test

**Figure 14:**
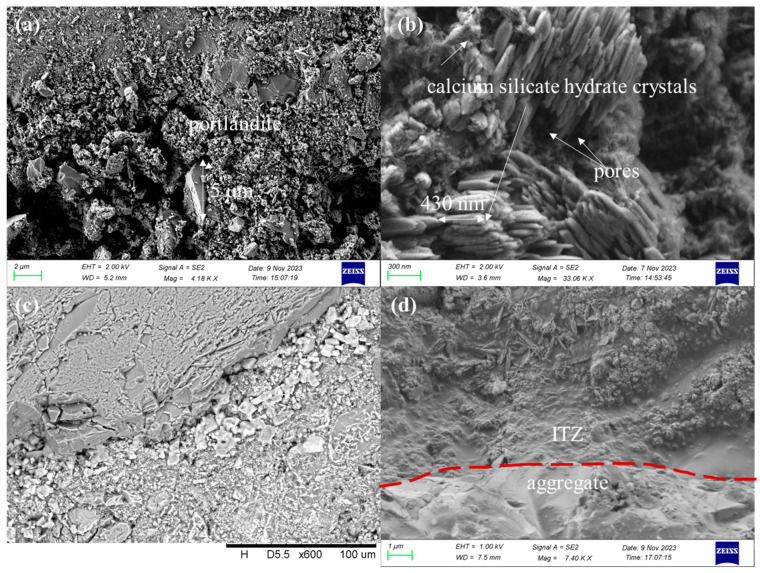
Microstructures of specimens after being exposed to 400 °C and recuring: (**a**) 400-0d, (**b**) 410-28d (**c**) 400-60d and (**d**) 410-60d.

The microstructures of specimens after being exposed to 400 °C and recuring can be observed in Figure 14.

In group 410, which was recured for 28 days, the SEM analysis showed the crystallization of calcium silicate hydrate (C-S-H) crystals at the interfacial transition zone (ITZ) with a preferred orientation, resulting in the formation of some pores. Previous studies have demonstrated that the preferred orientation of crystallization weakens the ITZ by creating a more porous microstructure [52]. However, the calcium silicate hydrate crystals identified in the thermogravimetric test (50–300 °C) exhibited a wide range of variability, making it difficult to discern them directly based on the TGA results. It has been reported that the addition of aluminum promotes the formation of larger and more ordered calcium silicate hydrate crystal structures [53]. Therefore, it is reasonable to assume that the addition of fly ash, which acts as an aluminum source, promotes the formation of larger and more oriented calcium silicate hydrate crystals in the ITZ compared with group 400. This, in turn, leads to the generation of a porous ITZ structure and contributes to the reduction in strength.

Figure 14c shows the SEM image of the group 400 sample recured for 60 days, obtained through BSE signals. Brighter phases adjacent to the ITZ likely correspond to portlandite, based on the imaging principle of BSE-SEM. The voids surrounding these phases indicate the damage caused by the expansive formation of portlandite, as discussed in Section 3.3.2. Furthermore, the SEM image in Figure 14d demonstrates the dense ITZ in the group 410 sample recured for 60 days, which provides an explanation for the observed increase in strength.

**Figure 15:**
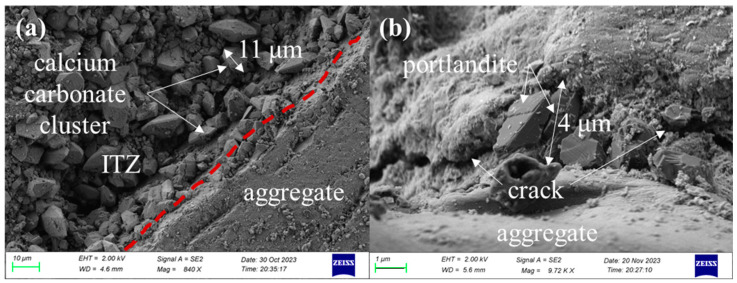
Microstructures of specimens after being exposed to 600 °C and recuring: (**a**) 600-60d and (**b**) 610-60d.

The microstructures of specimens after being exposed to 600 °C and recuring can be observed in Figure 15.

Significant formation of calcium carbonate crystals was observed in the ITZ of the group 600 samples after 60 days of recuring, providing supporting evidence for the results obtained from the DTG analysis. Figure 15a clearly illustrates the accumulation of large-sized calcium carbonate crystals, resulting in high porosity within the ITZ. This phenomenon can contribute to the reduction in strength observed in this group. The influence of fly ash as a crystallization-guiding agent, affecting the morphology of calcium carbonate, will be discussed in detail later in this section. Furthermore, the volume expansion of portlandite during crystallization contributes to the formation of microscopic cracks in both the ITZ and thematrix, as depicted in Figure 15b.

**Figure 16:**
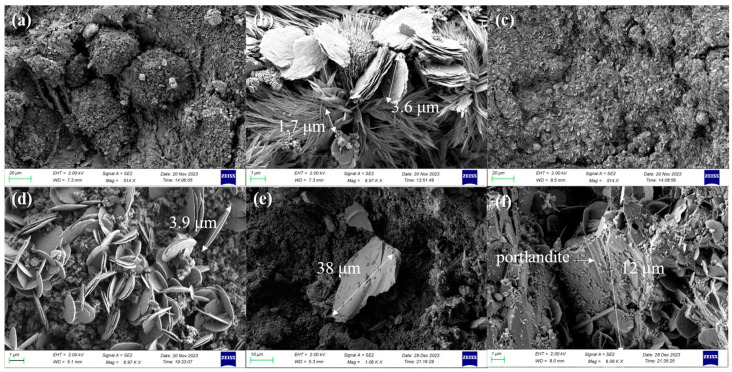
Microstructures of specimens after being exposed to 800 °C and recuring: (**a**,**b**) 800-28d; (**c**,**d**) 810-28d; (**e**) 800-28d; (**f**) 810-60d.

The microstructures of specimens after being exposed to 800 °C and recuring can be observed in Figure 16.

**Figure 17:**
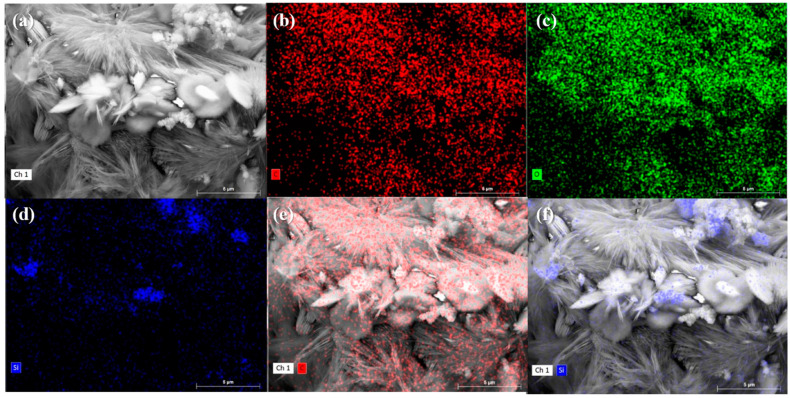
EDS mapping results of group 800-28d specimens: (**a**) SEM image of the crystals, (**b**) carbon, (**c**) oxygen, and (**d**) silicon. (**e**) Superimposed image of the SEM and EDS carbon distribution results. (**f**) Superimposed image of the SEM and EDS silicon distribution results.

The matrix microstructure of group 800 at 28 days of recuring, as illustrated in Figure 16a, exhibited intergrown crystals arranged in blocks with noticeable voids and gaps between them. These blocks displayed localized enlargement, and the presence of numerous needle-like crystals mixed with a smaller quantity of plate-like crystals was observed, as depicted in Figure 16b. To determine the composition of these crystals, we conducted EDS tests, and the results are presented in Figure 17 and Table 3. The mapping outcomes and quantitative analysis indicate that the needle-like crystals and plate-like crystals exhibit distinct morphological characteristics and are predominantly composed of calcium carbonate, with an extremely small amount of C-S-H gel present in the voids between these coarse crystals.

In contrast, with a recuring age of 28 days, the matrix microstructure of group 810 appeared relatively dense, as shown in Figure 16c. No crystal aggregation into blocks was observed. Similarly, localized magnification, shown in Figure 16d, revealed the significant presence of plate-like crystals interspersed with each other, and the voids between the crystals were filled with a considerable amount of C-S-H gel, resulting in a densified microstructure. However, no needle-like crystals were observed. The EDS mapping results of crystals with similar morphological features in group 800 strongly suggest that these lamellar plate-like crystals are also composed of calcium carbonate.

The abovementioned findings suggest that the addition of fly ash influenced the morphological characteristics of calcium carbonate. Specifically, the presence of fly ash inhibited the formation of needle-like calcium carbonate crystals. Previous studies have reported that metal cations, such as aluminum ions, can act as crystallization-guiding agents and alter the crystal morphology of calcium carbonate [54]. Therefore, it can be inferred that the observed morphological variation is primarily attributed to the release of metal cations from the metal elements present in the fly ash during the hydration process, which subsequently alters the crystallization process of calcium carbonate.

The observed change in crystal morphology facilitates the filling of voids between calcium carbonate crystals with C-S-H gel, leading to microstructural densification and improved material strength. These findings explain the lower strength growth in the 800 group compared with the 810 group at 28 days of recuring. The altered crystal morphology induced by fly ash promotes effective void filling, resulting in a denser microstructure and enhanced strength. The notable impact of this phenomenon following high-temperature exposure at 800 °C can be attributed to the substantial decomposition of calcium carbonate and subsequent rehydration during reconditioning, which are primarily observed following high-temperature exposures exceeding 750 °C.

Furthermore, Figure 16e reveals the absence of noticeable microcracks around portlandite, indicating the availability of ample space for its crystallization, as discussed in Section 3.3.2. Figure 16f illustrates that despite some surface coverage by calcium carbonate, the presence of hexagonal flake plate-like portlandite contributed to crack development.

#### 3.3.4. Nitrogen Adsorption Tests

**Figure 18:**
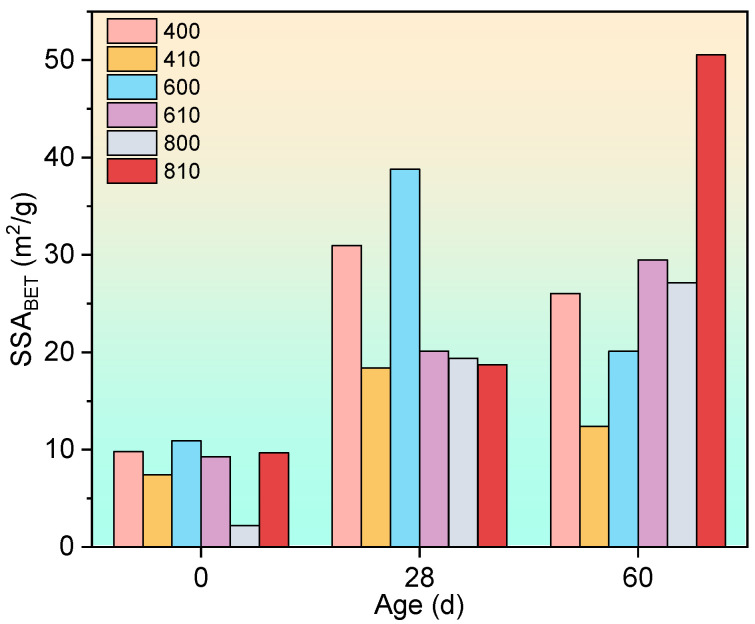
Specific surface area measured by N_2_-BET of all groups during the recuring process.

The N_2_-BET results are illustrated in Figure 18. According to the findings in the literature [15], the specific surface area measured by N_2_-BET (SSA_BET_) predominantly reflects the surface area of gel pores, as nitrogen sorption predominantly assesses the surface of gel pores. During the hydration process, it is typical to observe an initial increase followed by a decrease in SSA_BET_ as the microstructure of the cementitious material, particularly the C-S-H structures, undergoes densification [15].

In this study, both the anticipated “upward and then a downward trend” and continuous increments (observed during the initial stage) of SSA_BET_ were observed throughout the recuring process. Moreover, continuous increments were predominantly noted in the groups exposed to 600 °C and 800 °C, where a longer duration was required for the densification process due to more severe dehydration. Consequently, it is plausible to deduce that the rehydration process of the C-S-H structures was continuously ongoing at a microscopic scale, exerting little influence on the fluctuations in compressive strength.

## 4. Conclusions

In summary, the findings of this study provide valuable insights into the effects of fly ash on mortar strength during hydration and post-fire curing.

The presence of SiO_2_ in fly ash at 400 °C exhibited a pozzolanic reaction, consuming portlandite and positively influencing the mortar strength during further hydration under autoclaving conditions. Also, the negative effect of portlandite crystallization on strength was also suppressed by pozzolanic reaction during the recuring process.

However, it was observed that low substitution fly ash mortar became depleted of fly ash after a prolonged recuring age of 60 days after being exposed to 600 °C and 800 °C. This depletion hindered the inhibition of expansion crystallization of portlandite. The phenomenon of expansion crystallization, coupled with the development of a dense microstructure after 60 days, led to the generation of numerous microcracks, resulting in a decrease in mortar strength at this stage.

Furthermore, the aluminum content in fly ash caused the oriented precipitation of coarse-grained C-S-H crystals, which weakened the ITZ. However, this effect on strength was primarily prominent after high-temperature exposure at 400 °C. Additionally, after exposure to 800 °C, the decomposition of calcium carbonate occurred, and the aluminum elements in fly ash acted as crystallization-guiding agents during rehydration, affecting the morphology of the calcium carbonate crystals. This alteration facilitated the easier filling of pores by the C-S-H gel, resulting in densification of the microstructure and positively impacting the strength recovery.

These findings highlight the complex interplay between fly ash properties, temperature exposure, and curing duration in determining the strength characteristics of mortars. Understanding these relationships is crucial for optimizing the use of fly ash in concrete applications, particularly in post-fire scenarios.

## Figures and Tables

**Table 1:** Mix ratio (kg).

Group Name ^a^	Cement	Fly Ash	Water	Fine Aggregate
X00	100	0	50	300
X10	90	10	50	300

^a^ Note: X indicates the exposure temperature (°C)/100 and the last two numbers indicate the replacing rate, e.g., group 800 indicates the group exposed to 800 °C with no fly ash as the binder material; group 010 indicates the group without high-temperature exposure and with 10% of the binder materials replaced by fly ash.

**Table 2:** Chemical composition of cement and fly ash (wt%).

Type	SiO_2_	Al_2_O_3_	K_2_O	CaO	Fe_2_O_3_	SO_3_	MgO	Others
Cement	15.08	3.73	0.92	70.45	3.10	4.06	1.80	0.86
Fly ash	66.48	14.92	3.32	5.41	9.86	-	-	-

**Table 3:** EDS quantitative analysis results.

Element	O	C	Ca	Si
Atom percentage (%)	44.11	20.88	19.43	5.17

## Data Availability

Data are contained within the article.
